# Probiotic *Escherichia coli* Nissle 1917 protect chicks from damage caused by *Salmonella enterica* serovar Enteritidis colonization

**DOI:** 10.1016/j.aninu.2023.06.001

**Published:** 2023-06-08

**Authors:** Shu Wu, Qianyun Zhang, Guanglei Cong, Yunqi Xiao, Yiru Shen, Shan Zhang, Wenchang Zhao, Shourong Shi

**Affiliations:** Department of Feed and Nutrition, Poultry Institute, Chinese Academy of Agricultural Sciences, Yangzhou, China

**Keywords:** *S.* Enteritidis, Gut microbiota, Nissle 1917, Angiotensin-converting enzyme 2–solute carrier family 6-member 19 pathway

## Abstract

As a foodborne pathogen of global importance, *Salmonella enterica* serovar Enteritidis (*S.* Enteritidis) is a threat to public health that is mainly spread by poultry products. Intestinal Enterobacteriaceae can inhibit the colonization of *S.* Enteritidis and are regarded as a potential antibiotic substitute. We investigated, in chicks, the anti-*S.* Enteritidis effects of *Escherichia coli* (*E. coli*) Nissle 1917, the most well-known probiotic member of Enterobacteriaceae. Eighty 1-d-old healthy female AA broilers were randomly divided into 4 groups, with 20 in each group, namely the negative control (group P), the *E. coli* Nissle 1917-treated group (group N), the *S*. Enteritidis-infected group (group S) and the *E. coli* Nissle 1917-treated and *S*. Enteritidis-infected group (group NS). From d 5 to 7, chicks in groups N and NS were orally gavaged once a day with *E. coli* Nissle 1917 and in groups P and S were administered the same volume of sterile PBS. At d 8, the chicks in groups S and NS were orally gavaged with *S*. Enteritidis and in groups P and N were administered the same volume of sterile PBS. Sampling was conducted 24 h after challenge. Results showed that gavage of *E. coli* Nissle 1917 reduced the spleen index, *Salmonella* loads, and inflammation (*P* < 0.05). It improved intestinal morphology and intestinal barrier function (*P* < 0.05). *S.* Enteritidis infection significantly reduced mRNA expression of angiotensin-converting enzyme 2 (*ACE2*) and solute carrier family 6-member 19 (*SLC6A19*) in the cecum and the content of Gly, Ser, Gln, and Trp in the serum (*P* < 0.05). Pretreatment with *E. coli* Nissle 1917 yielded mRNA expression of *ACE2* and *SLC6A19* in the cecum and levels of Gly, Ser, Gln, and Trp in the serum similar to that of uninfected chicks (*P* < 0.05). Additionally, *E. coli* Nissle 1917 altered cecum microbiota composition and enriched the abundance of *E. coli*, Lactobacillales, and Lachnospiraceae. These findings reveal that the probiotic *E. coli* Nissle 1917 reduced *S.* Enteritidis infection and shows enormous potential as an alternative to antibiotics.

## 1 Introduction

*Salmonella*, as an important foodborne pathogen in the world, has caused a great quantity of human deaths and economic losses in animal husbandry. Over one million foodborne salmonellosis cases were reported in the European Union in 2017 (EFSA and ECDC, 2018) and 59,841 were reported in the America in 2018 (Tack et al., 2019). *Salmonella enterica* serovar Enteritidis is one of the common frequent serotypes isolated from these clinical cases, accounting for about 40% to 60% of foodborne salmonellosis worldwide each year (Pearce et al., 2018). Poultry species are the primary host of *S.* Enteritidis and contaminated poultry-derived products have been identified as a major source of *S.* Enteritidis infections in humans (Mahmoud, 2012). The popular of *S.* Enteritidis in chicken meat is associated with the morbidity of human caused by this serotype (Shah et al., 2017). These phenomena emphasize the significance of studying *S.* Enteritidis infection in poultry for both public health and poultry production concerns.

Probiotics can change the intestinal microbes in poultry and play a role in the competitive exclusion of *Salmonella*, thereby increasing the resistance of chicks to colonization by *Salmonella* (Lan et al., 2020). Host commensal Enterobacteriaceae are the key species affecting the susceptibility differences to *Salmonella* (Velazquez et al., 2019) and resistance to intestinal colonization by *S.* Enteritidis by competing for oxygen (Litvak et al., 2019). *Escherichia coli* Nissle 1917 is a non-pathogenic strain that was firstly isolated from a man who appeared to be resistant to a diarrhea outbreak (Nissle et al., 1959). Nissle 1917 can establish lasting colonization in the intestine and is used to treat or prevent various intestinal disorders (Cukrowska et al., 2002; Kruis et al., 2004), including the colonization of pathogens (Deriu et al., 2013). Nissle 1917 also has been shown to be beneficial for poultry, such as enhanced early intestinal maturation of young turkey poults (Moyle et al., 2012), reduced shedding of enteric pathogens, higher body weight, and modulating the stress response of poultry in respiratory tract attack of avian pathogenic *E. coli* (Huff et al., 2006). These studies mainly focused on turkey, and little is known about the effects of Nissle 1917 on the resistance of chicks to *S.* Enteritidis colonization and infection.

Angiotensin-converting enzyme 2 (ACE2), well-known as the functional receptor for coronaviruses (Li et al., 2003; Walls et al., 2020), was initially found to be a strong negative regulator of the renin-angiotensin system (RAS) and plays a vital role in many physiological processes (Vickers et al., 2002; Mendoza-Torres et al., 2015; Passos-Silva et al., 2015). Previous studies have reported that the expression of *ACE2* in intestinal epithelial cells is associated with animal intestinal health (Zhang et al., 2020). *ACE2* is necessary for intestinal expression of the neutral amino acid transporter, solute carrier family 6 member 19 (SLC6A19)/B^0^AT1 (Kowalczuk et al., 2008), which can affect the microecological balance of the intestinal tract and regulate intestinal inflammation by regulating neutral amino acid transport (Camargo et al., 2009). Knockout of *ACE2* significantly increased the susceptibility of the host to intestinal inflammatory diseases (Hashimoto et al., 2012). However, the relationship between *ACE2* expression and the inflammation induced by *S.* Enteritidis infection remains unknown. More importantly, a previous study by our laboratory found that the *ACE2* gene is highly expressed in chicken intestinal tissues (Cong et al., 2021). Therefore, we sought to explore whether *ACE2* mediated intestinal inflammation in chicken that resulted from *S*. Enteritidis infection, and further investigated whether Nissle 1917 helped chicks resist *S*. Enteritidis infection by up-regulating *ACE2* expression.

## 2 Materials and methods

### 2.1 Animal ethics statement

All animal experiments were conducted according to the Regulations of the Experimental Animal Administration issued by the State Committee of Science and Technology of the People's Republic of China and approved by the Animal Care and Use Committee of the Poultry Institute, Chinese Academy of Agriculture Science (No. CNP20210402).

### 2.2 Animals and management

Female 1-d-old (AA) broiler chickens were purchased from Jiangsu Jinghai Poultry Industry Group Co., Ltd., Nantong, Jiangsu, China. After hatching, cloacal swab tests were carried out immediately to exclude *Salmonella* infection (Sabry et al., 2020). Broiler chickens were raised in wire-screened cages. They had free obtained to water and food under a 24-h light photoperiod. During the first week, the temperature in the broiler house ranged from 32 to 35 °C, then decreased by 1 °C/d until reaching the final temperature of 30 °C on d 9. During the feeding period, feed a *Salmonella*-free diets without antibiotics or anticoccidial drugs, and the diets was formulated to meet or slightly exceed all nutrient requirements, according to the nutrient specification for Arbor Acres Broiler Management Handbook (Aviagen, 2018) (Table S1).

### 2.3 Culture of *E. coli* Nissle 1917 and *S.* Enteritidis

*E. coli* Nissle 1917 (bio-089,089) was purchased from Biobw Biotechnology Co., Ltd., Beijing, China. Before gavaging, the Nissle 1917 strain was grown in Luria–Bertani broth (Qingdao–Hope Biotechnology Co., Ltd., Qingdao, Shandong, China) overnight at 37 °C with constant shaking. The bacteria were harvested by centrifugation at 7155 × *g* at 4 °C for 10 min, washed twice with sterile phosphate-buffered saline (PBS), and then re-suspended to a concentration of 4 × 10^8^ CFU/mL, according to plate counting on MacConkey agar (Qingdao–Hope Biotechnology Co., Ltd.).

The *S.* Enteritidis strain CMCC(B)50,041 was purchased from Suzhou Beina Chuanglian Biotechnology Co., Ltd., Jiangsu, China and cultured as previously described (Wu et al., 2021). Briefly, before challenging, the *S.* Enteritidis was grown in advanced Martin broth (Qingdao Hope Bio-Technology Co., Ltd., Shandong, China) overnight at 37 °C with constant shaking. The bacteria were harvested by centrifugation at 7155 × *g* at 4 °C for 10 min, washed twice with sterile PBS, and re-suspended to a concentration of 2.5 × 10^9^ CFU/mL, according to plate counting on xylose lysine desoxycholate (XLD) agar (Qingdao–Hope Biotechnology Co., Ltd.).

### 2.4 Gavage of Nissle 1917 for *S.* Enteritidis infected or uninfected chicks

Eighty 1-d-old healthy female AA broilers were randomly divided into 4 groups. The treatment groups were: (1) negative control (no Nissle 1917 treatment and no *S*. Enteritidis infection, group P); (2) the Nissle 1917-treated group (group N); (3) the *S.* Enteritidis-infected group (group S); and (4) the Nissle 1917-treated and *S.* Enteritidis-infected group (group NS). All chicks were fed a basal diet. On d 5 to 7, chicks in groups N and NS were orally gavaged once a day with 0.4 mL of a suspension of 4 × 10^8^ CFU/mL *E. coli* Nissle 1917; the chicks in groups P and S were administered the same volume of sterile PBS. At d 8, the chicks in groups S and NS were orally gavaged with a 0.4 mL *S.* Enteritidis strain suspension at a dose of 2.5 × 10^9^ CFU/mL; the chicks in groups P and N were administered the same volume of sterile PBS.

### 2.5 Sample collection

At 24 h post-infection, 6 broilers at 9 d old were randomly collected from each group, weighed, and euthanized by exsanguination from the jugular vein. Individual blood samples were harvested from the jugular vein, and serum samples were separated by centrifugation at 3000 × *g* at 4 °C for 10 min and stored at −80 °C for further analysis. The liver and spleen were collected and weighed for tissue index calculations. Next, 0.2 to 0.4 g of liver, spleen and cecal chyme were collected aseptically and stored at 4 °C for bacteria load quantification. Small segments (1 to 2 cm) of the liver and mid-jejunum were collected and fixed in a 10% formaldehyde solution for histopathological examination. The remaining cecal content was collected, frozen in liquid nitrogen, and stored at −80 °C for 16S rRNA sequencing analysis. The cecal tonsil and the jejunum tissues were collected, frozen in liquid nitrogen, and stored at −80 °C for measuring target gene mRNA levels.

### 2.6 Bacteria load measurements

To evaluate *S.* Enteritidis loads in the tissues, as well as *E. coli* loads in the cecum chyme, samples were weighed and diluted to 10% in sterile PBS. Samples were homogenized using a SCIENTZ-48 homogenizer (Ningbo Xingzhi Biotechnology Co., Ltd., Ningbo, China) at 60 Hz for 120 s. The homogenate liquid of the cecum chyme was 10-fold diluted serially. Aliquots (50 μL) from each sample were transferred to XLD (for *S.* Enteritidis) or MacConkey (for *E. coli*) agar and incubated for 24 h at 37 °C. The bacteria counts were calculated as lg CFU/g of tissue or cecum chyme.

### 2.7 Liver histopathology and intestinal morphology determination

Broiler tissue histopathology and intestinal morphology were determined as previously described (Hu et al., 2020). Briefly, small segments of the liver and middle jejunum were dehydrated using an ascending ethanol gradient after being fixed in 10% buffered formaldehyde (pH 7.2). After xylene clearing, the samples were embedded in paraffin and cut into 5-μm-thick slices, which were then mounted and stained with hematoxylin and eosin (H&E). Inflammatory infiltration and general damage in the liver, as well as the villus height (VH), crypt depth (CD), and muscle thickness (MT) of the jejunum, were measured under a fluorescence microscope (DM4000B, Leica Microsystems, Wetzlar, Germany). Ten well-oriented and intact villi and 10 crypts per sample were measured (Qaisrani et al., 2014), and the ratio of VH-to-CD (VCR) was calculated.

### 2.8 RNA isolation and quantitative real-time PCR

According to the manufacturer's instructions, total RNA was isolated from the cecal tonsils and jejunum tissues of broilers using RNAsimple Total RNA Kit (DP419, Tiangen Biotech Co., Ltd., Beijing, China). NanoDrop 2000 spectrophotometer (Thermo Fisher Scientific, Rockford, IL, USA) was using to measure the RNA concentration and purity, and 1.5% agarose gel electrophoresis was using to assess the RNA quality. After that, according to the manufacturer's instructions, use FastKing gDNA Dispelling RT SuperMix Kit (KP118, Tiangen Biotech Co., Ltd., Beijing, China) to reverse transcribe total RNA. Reverse transcription was performed at 42 °C for 15 min, followed by thermal inactivation at 95 °C for 3 min. The cDNA was stored at – 20 °C until further use. The SuperReal PreMix Plus (SYBR Green) Kit (FP205, Tiangen Biotech Co., Ltd., Beijing, China) in StepOnePlusTM Real-Time PCR System (Applied Biosystems, Foster City, CA, USA) was used for real-time fluorescence quantitative technique (qRT-PCR) according to the optimized PCR protocol. The scheme consists of an initial denaturing step at 95 °C for 15 min, followed by 40 cycles of 10 s denaturing cycles at 95 °C, and 30 s annealing/extending at 60 °C, and finally at 95 °C for 15 s. Five housekeeping genes were tested, and then the most stable housekeeping gene in the cecal tonsil or jejunum samples was selected. Due to high expression stability, glyceraldehyde-3-phosphate dehydrogenase (GAPDH) was eventually chosen to normalize gene expression. The efficiency of all tested genes was between 90% and 110%. Target gene expression was normalized with GAPDH gene expression. The mRNA expression was calculated by the 2^−ΔΔCt^ method (Livak and Schmittgen, 2001). The primers for inducible nitric oxide synthase (*NOS2*), interferon-gamma (*IFN-γ*), tumor necrosis factor-alpha (*TNF-α*), interleukin 1 beta (*IL-1β*), *IL-6, IL-8, IL-10*, occludin, claudin-1, zonula (*ZO-1*), mucin 2 (*MUC2*), *ACE2*, *SLC6A19*, and *GAPDH* are listed in Table S2.

### 2.9 DNA extraction and sequencing library construction

Genomic DNA was extracted from homogenized cecal chyme using the Stool DNA Kit (DP712, Tiangen Biotech Co., Ltd., Beijing, China) and stored at −20 °C. DNA concentration and purity were determined by a NanoDrop 2000 spectrophotometer, and DNA quality was assessed by 2% agarose gel electrophoresis. The V4 region of the bacterial 16S rRNA gene was PCR amplified using the barcoded primers 515 F (5′-GTGCCAGCMGCCGCGGTAA-3′) and 806 R (5′-GGACTACHVGGGTWTCTAAT-3′) (Yang et al., 2017). Amplicons with 400 to 450 bp were extracted and used for further analysis (Caporaso et al., 2011; Gao et al., 2017). PCR products were purified using the QIAquick Gel Extraction Kit (Qiagen Inc., Santa Clara, CA, USA). Sequencing libraries were generated using the Illumina TruSeq DNA PCR-Free Sample Preparation Kit (Illumina, San Diego, CA, USA) following the manufacturer's recommendations. After Qubit-based quantification and library qualification, the library was subjected to sequencing at Novogene Co., Ltd., Beijing, China, using the Illumina NovaSeq6000 platform.

### 2.10 Quality filtering and sequence analysis

Raw Illumina 250-bp paired-end reads were trimmed of barcodes and primers and combined using Flash software (V1.2.7) with default parameters (Magoč and Salzberg, 2011). The obtained raw tags were quality-filtered using QIIME V1.9.1 to obtain effective tags (Caporaso et al., 2010). All effective tags were clustered into operational taxonomic units (OTUs) with 97% homology similarity by Uparse V7.0.1001 (Edgar, 2013). OTU taxonomic information was annotated by RDP Classifier using a 0.8 to 1 confidence threshold for taxonomic assignment (Wang et al., 2007). Alpha and beta diversity and the significance of taxonomic differences between samples were estimated by QIIME (V1.9.1) (Zhang et al., 2018) and linear discriminant analysis effect size (LEfSe) (Zhang et al., 2020) as previously described.

### 2.11 Serum neutral amino acid analyses

Concentrations of serum amino acids, except tryptophan, were determined as previously described (Espe et al., 2014). In brief, serum samples were oxidized using a hydrogen peroxide solution (containing phenolic formic acid) at 0 °C for 16 h, and sodium pyrosulfite was added after the reaction to decompose the excess peroxyformic acid. Subsequently, 6 mol/L HCl solution was added to the oxidized samples and hydrolyzed at 110 °C for 23 h to release free amino acids. The concentrations of free amino acids were then determined on an amino acid analyzer (Biochrom, Version 30, Biochrom Ltd., Cambridge, UK) equipped with an ion-exchange column. Tryptophan was determined by a high-performance liquid chromatography (HPLC; Shimadzu, Tokyo, Japan). In brief, samples were saponified under alkaline conditions with barium hydroxide solution in the absence of air at 110 °C for 20 h in an autoclave. After adjusting the hydrolysate pH to 3.0, the tryptophan was separated by reversed-phase chromatography RP-18 on a HPLC column, and the chromatograms were integrated using Labsolutions software with a fluorescence detector (Shimadzu, Tokyo, Japan).

### 2.12 Co-culture assay of *S.* Enteritidis and Nissle 1917

To ascertain the in vitro effect of Nissle 1917 on *S.* Enteritidis, the LB broths containing 10^6^ CFU/mL of *S.* Enteritidis or Nissle 1917 were mixed 1:1 and incubated at 37 °C for 24 h. The LB broth containing 10^6^ CFU/mL of *S.* Enteritidis without Nissle 1917 was assigned as the control group (Velazquez et al., 2019). Then, 50 μL of the bacterial suspensions was plated on XLD agar and incubated for 24 h at 37 °C. The number of *S.* Enteritidis were counted, and the fold change between the 2 groups was calculated according to the following equation: Fold change = CFU of mixed group/Mean CFU of group Control.

### 2.13 Statistical analysis

Statistical analyses were carried out with SPSS for Windows V22.0 (SPSS Inc., Chicago, IL, USA). Differences among different groups were analyzed by one-way ANOVA and the Wilcox rank-sum test. The specific analyzing method of each data was shown in the corresponding Figure legend or Table note. Data are expressed as mean ± SEM. A *P*-value < 0.05 is statistically significant.

## 3 Results

### 3.1 *E. coli* Nissle 1917 protected the host against *S.* Enteritidis infection

**Fig. 1:**
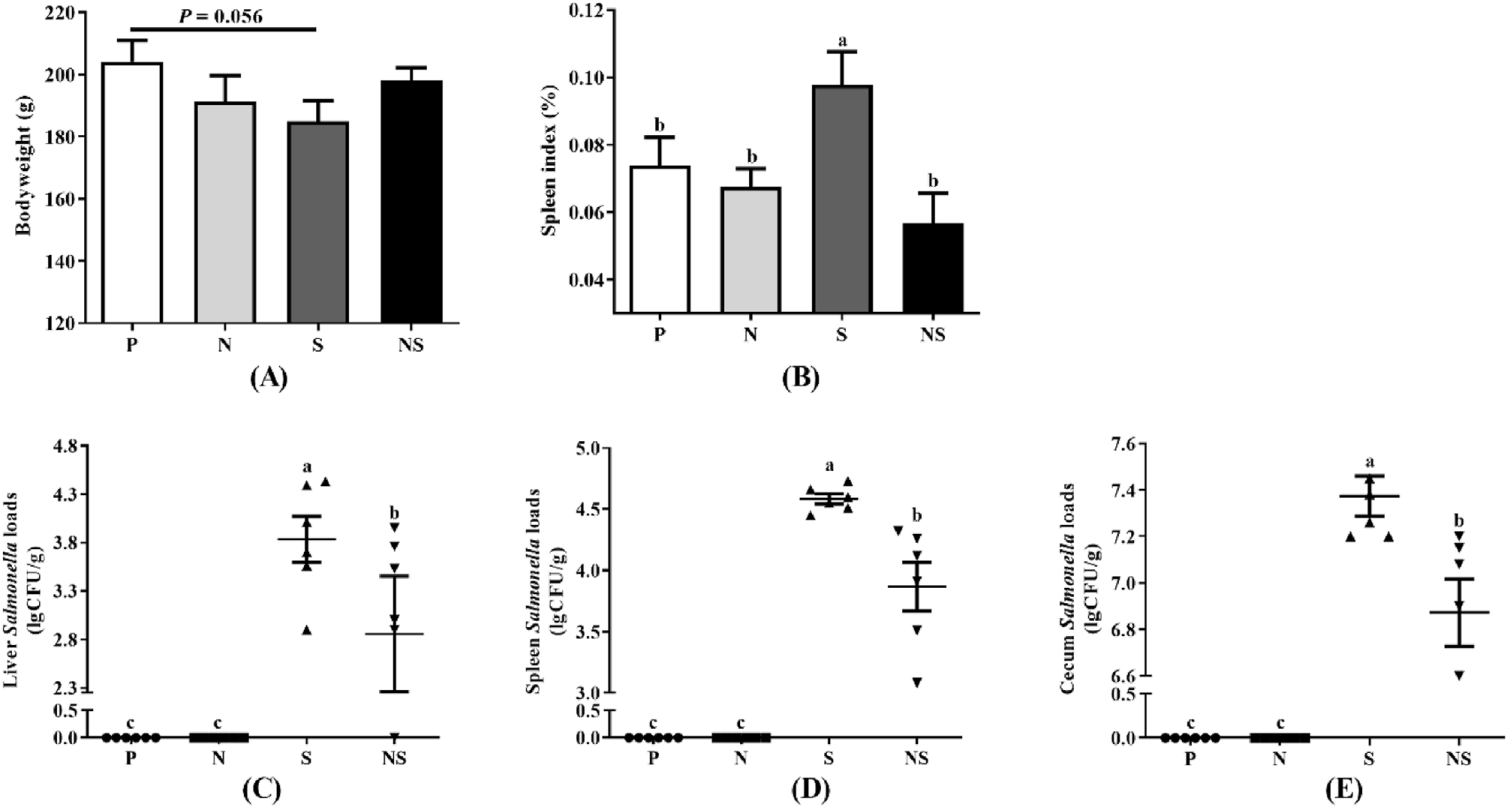
Bodyweight, spleen index and *Salmonella* loads of infected chicks. (A) Bodyweight. (B) Spleen index. (C) *Salmonella* loads in liver. (D) *Salmonella* loads in spleen. (E) *Salmonella* loads in cecum chyme. Group P = uninfected chicks; Group N = chicks treated with Nissle 1917 alone; Group S = *S*. Enteritidis infected chicks; Group NS = pretreated with Nissle 1917 + *S*. Enteritidis infected chicks. Data were analyzed by one-way ANOVA and shown as means ± SEM (*n* = 6). Bars with different letters are significantly different among different groups.

To verify the role of *E. coli* Nissle 1917 on host resistance to *S.* Enteritidis infection, chicks were inoculated with Nissle 1917 three times by oral gavage before *S.* Enteritidis infection. The body weight, liver index, spleen index, *S.* Enteritidis invasion of the liver and spleen, and *S.* Enteritidis colonization in cecum chyme were determined (Fig. 1). Compared to the group P, the body weight of group S were decreased (*P* = 0.056), while that of group N and group NS were not changed. The spleen index of chicks in group S was significantly higher than that of chicks in the other 3 groups (*P* < 0.05). Pretreatment with Nissle 1917 reduced *Salmonella* loads in the liver, spleen, and cecum chyme of infected chicks by 9.33, 5.13, and 4.90, respectively (*P* < 0.05).

### 3.2 *E. coli* Nissle 1917 alleviated inflammation

**Fig. 2:**
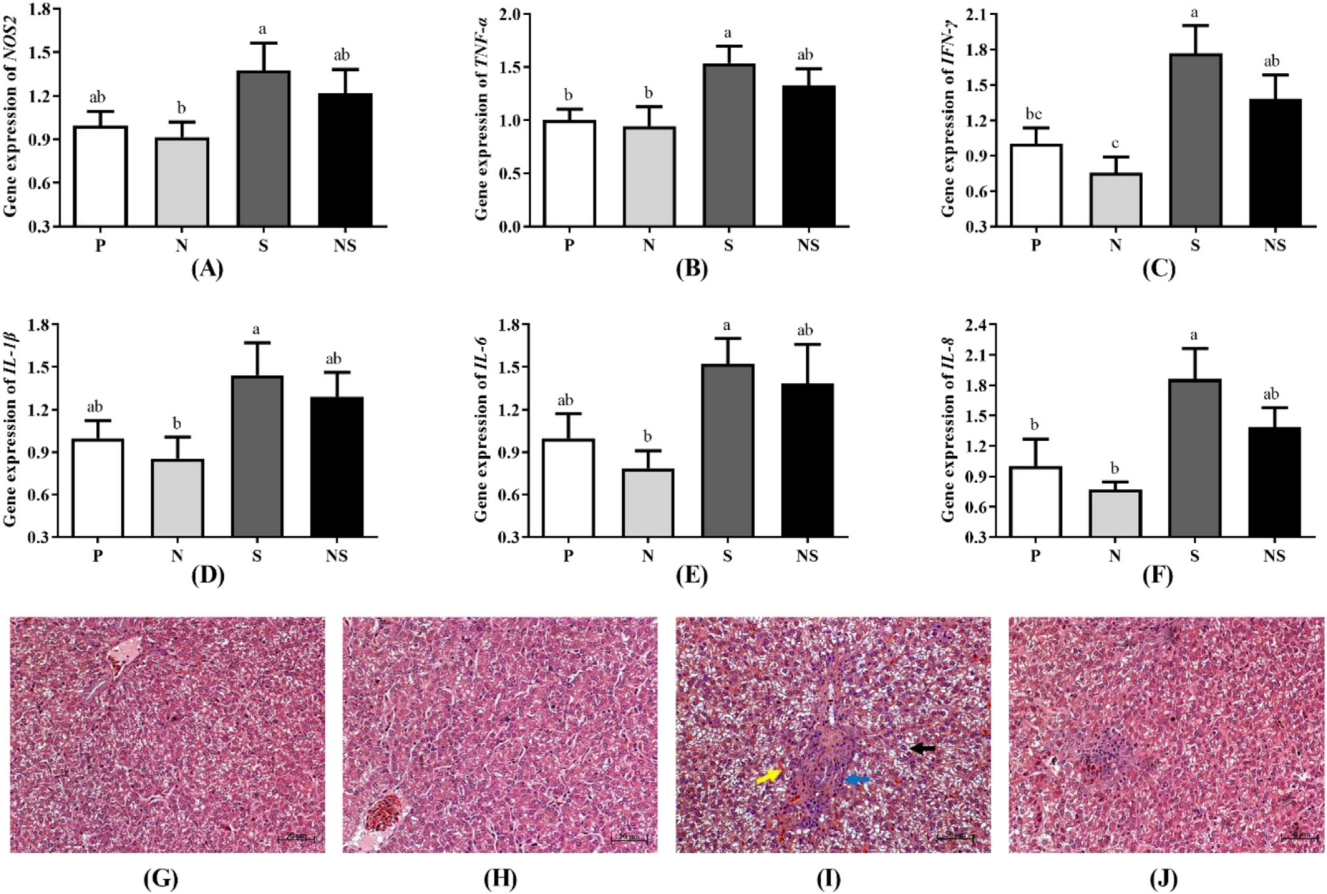
Effects of Nissle 1917 on the inflammation response of *S.* Enteritidis infected or uninfected chicks. (A–F) Relative expression of genes coding for pro-inflammatory cytokines *NOS2* (A), *TNF-α* (B), *IFN-γ* (C), *IL-1β* (D), *IL-6* (E) and *IL-8* (F) in the cecal tonsils. Group P = uninfected chicks; Group N = chicks treated with Nissle 1917 alone; Group S = *S*. Enteritidis infected chicks; Group NS = pretreated with Nissle 1917 + *S*. Enteritidis infected chicks. Data were analyzed by one-way ANOVA and shown as means ± SEM (*n* = 6). Bars with different letters are significantly different among different groups. (G–J) Representative liver histopathology of chicks in group P (G), group N (H), group S (I), and group NS (J) (H&E staining, the black arrow indicates vacuoles, the blue arrow indicates lymphocytic nodules, and the yellow arrow indicates heterophilic cell infiltration). Magnification, × 200. Scale bar = 50 μm.

Gene expression of *NOS2, TNF-α, IFN-γ, IL-1β, IL-6,* and *IL-8* in the cecal tonsils of chicks in each group is displayed in Fig. 2A–F. Specifically, *S.* Enteritidis infection induced up-expression of 3 pro-inflammatory cytokines *TNF-α, IFN-γ,* and *IL-8* genes in the cecal tonsil of chicks (*P* < 0.05), which returned to levels similar to uninfected chicks when pretreated with Nissle 1917. The expression of all tested pro-inflammatory genes in *S.* Enteritidis-infected chicks was significantly higher than that in chicks treated with Nissle 1917 alone. Chicks of group S showed obvious liver histological lesions with more vacuoles, lymphocytic nodules, and heterophilic cell infiltration (Fig. 2I). Low lymphocytic cell infiltration was observed in chicks of group NS (Fig. 2J). No liver pathological changes were observed in chicks of groups N and P (Fig. 2G and H).

### 3.3 *E. coli* Nissle 1917 improved intestinal morphology and intestinal barrier function

**Fig. 3:**
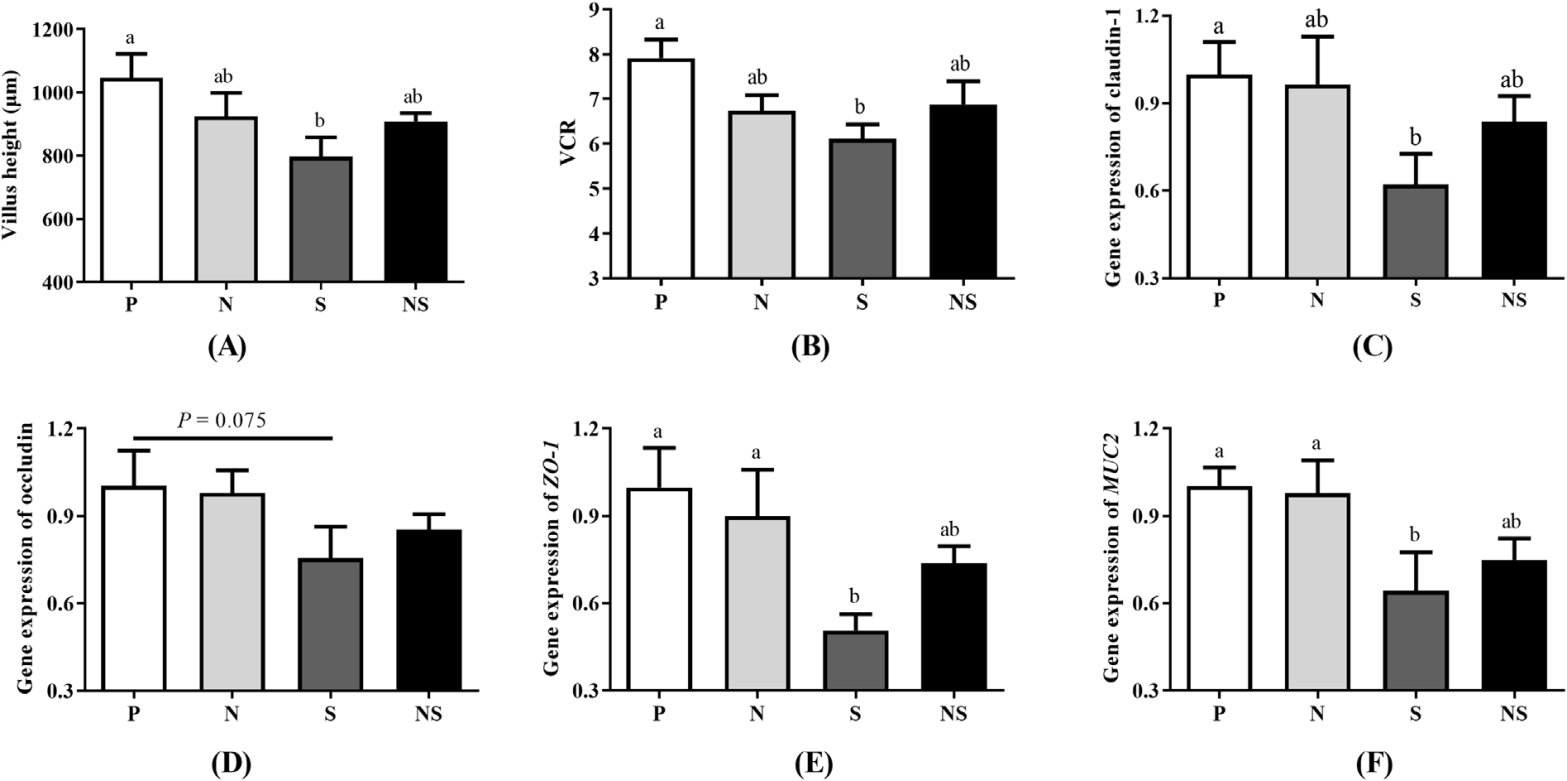
Effects of Nissle 1917 on the jejunum morphology and intestinal barrier function of *S.* Enteritidis infected vs. uninfected chicks. (A) Villus height. (B) Ratio of villus height to crypt depth (VCR). (C–F) Relative expression of genes coding for tight junction proteins claudin-1 (C), occludin (D), *ZO-1* (E), and *MUC2* (F) in the jejunum. Group P = uninfected chicks; Group N = chicks treated with Nissle 1917 alone; Group S = *S*. Enteritidis infected chicks; Group NS = pretreated with Nissle 1917 + *S*. Enteritidis infected chicks. Data were analyzed by one-way ANOVA and shown as means ± SEM (*n* = 6). Bars with different letters are significantly different among different groups.

Both *Salmonella* infection and treatment with Nissle 1917 showed no significant effects on jejunal CD and MT (Fig. S1). *Salmonella* infection decreased VH and the VCR in the jejunum (*P* < 0.05), which returned to levels similar to uninfected chicks when pretreated with Nissle 1917 (Fig. 3A and B). Intestinal barrier function among the 4 groups was compared by determining the expression of the claudin-1, occludin, *ZO-1*, and *MUC2* genes in the jejunum. As shown in Fig. 3C–F, compared with the uninfected chicks, expression of claudin-1, *ZO-1,* and *MUC2* in the jejunum was decreased by *S.* Enteritidis infection, which returned to levels similar to uninfected chicks when pretreated with Nissle 1917 (*P* < 0.05). The expression of occludin showed a similar trend as the other 3 genes, although the differences among groups were not statistically significant (*P* = 0.075).

### 3.4 *E. coli* Nissle 1917 improved gene expression in cecal tissue of *ACE2* and *SLC6A19* as well as the content of neutral amino acids in serum

**Fig. 4:**
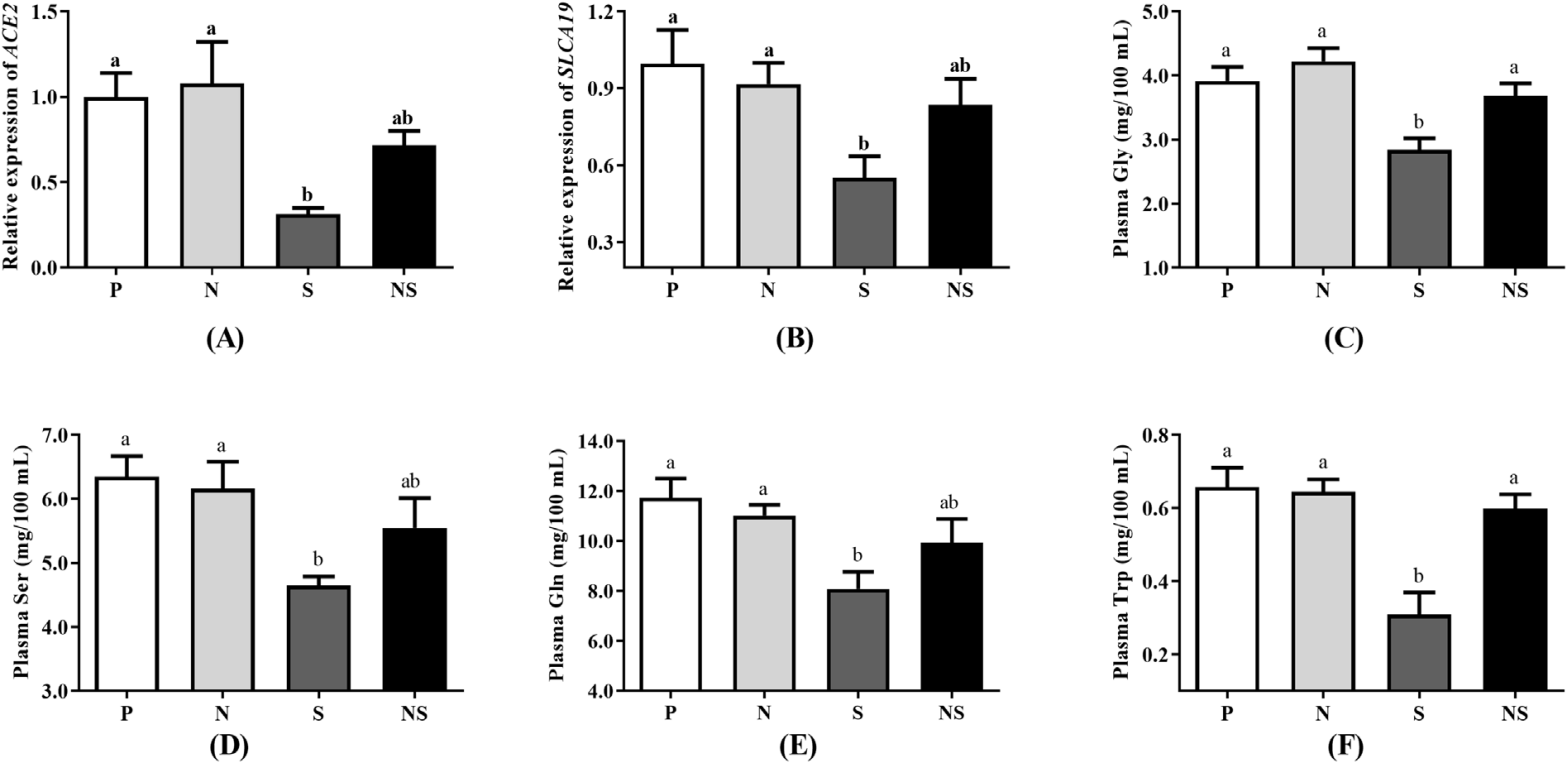
Effects of Nissle 1917 on gene expression of *ACE2* and *SLC6A19* and the content of neutral amino acids of *S.* Enteritidis infected vs. uninfected chicks. (A) Relative expression of *ACE2* in the cecum. (B) Relative expression of *SLCA19* in the cecum. (C) Content of glycine (Gly) in the plasma. (D) Content of serine (Ser) in the plasma. (E) Content of glutamine (Glu) in the plasma. (F) Content of tryptophan (Trp) in the plasma. Group P = uninfected chicks; Group N = chicks treated with Nissle 1917 alone; Group S = *S*. Enteritidis infected chicks; Group NS = pretreated with Nissle 1917 + *S*. Enteritidis infected chicks. Data were analyzed by one-way ANOVA and shown as means ± SEM (*n* = 6). Bars with different letters are significantly different among different groups.

To study whether the ACE2-SLC6A19 pathway mediated the anti-inflammatory effect of Nissle 1917 in *S.* Enteritidis infected chicks, mRNA expression of *ACE2* and *SLC6A19* in the cecum of chicks was analyzed. The results revealed that *S.* Enteritidis infection significantly reduced mRNA expression of *ACE2* and *SLC6A19* in the cecum (*P* < 0.05), while pretreatment with Nissle 1917 yielded mRNA expression of *ACE2* and *SLC6A19* in the cecum similar to that of uninfected chicks (Fig. 4A and B). In addition, the content of neutral amino acids in serum were also analyzed. As expected, the results showed that *S.* Enteritidis infection significantly reduced the content of 4 neutral amino acids (Gly, Ser, Gln, and Trp) in serum, whereas pretreatment with Nissle 1917 restored the content of these plasma neutral amino acids (Fig. 4C–F).

### 3.5 *E. coli* Nissle 1917 altered cecum microbiota composition and enriches the abundance of *E. coli*

**Fig. 5:**
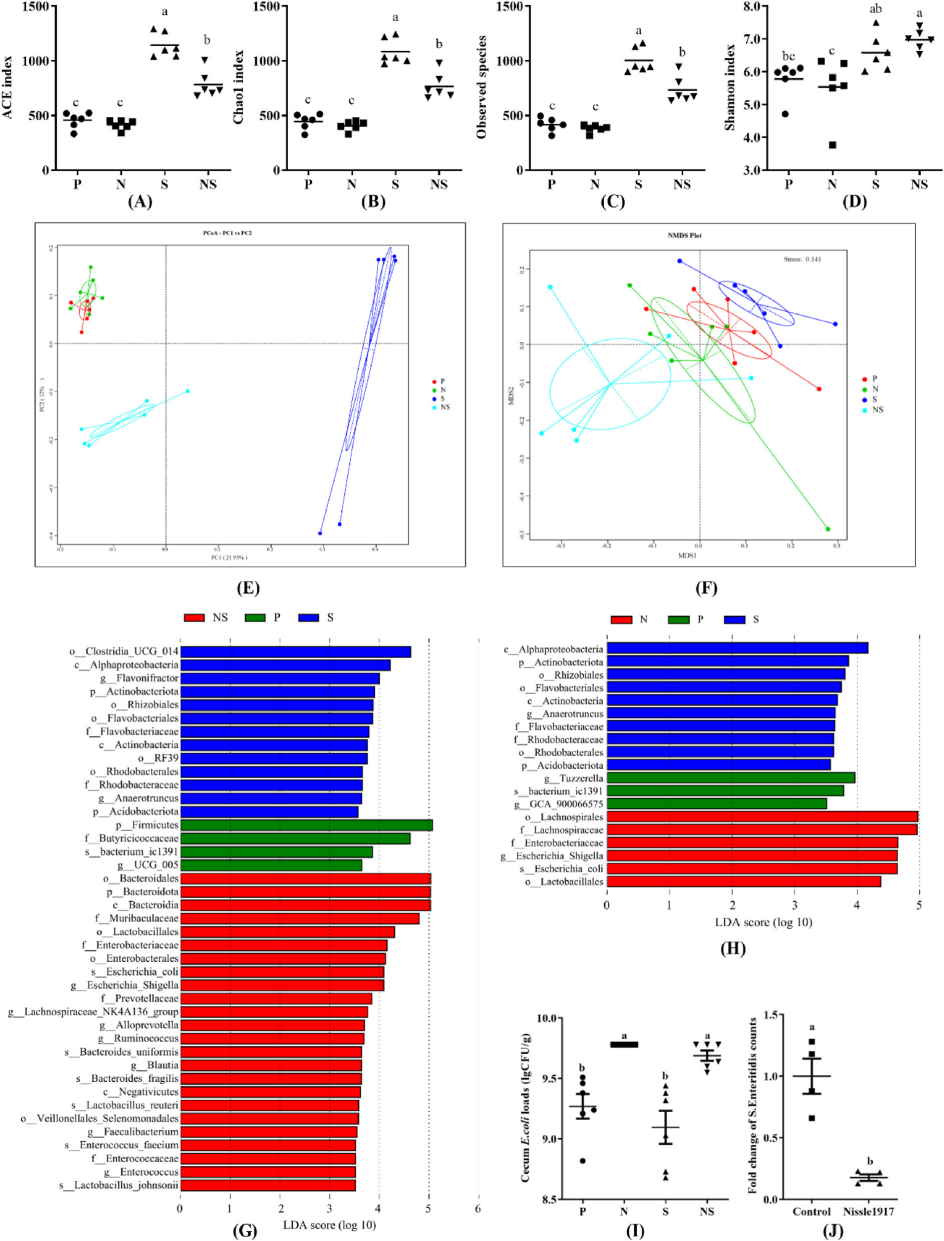
Effects of Nissle 1917 on cecum microbiota composition of chicks uninfected vs. infected with *S.* Enteritidis. (A–D) Alpha diversity analyzed by ACE (A), Chao1 (B), Observed_species (C) and Shannon (D) indices. Data are shown as means ± SEM (*n =* 6). Boxes with different letters are significantly different among different groups. (E–F) Beta diversity analyzed by PCoA (E) and NMDS (F). (G–H) LEfSe analysis of cecum microbiota among the groups NS-vs-S-vs-P (G) and groups N-vs-S-vs-P (H). Red bars are taxa enriched in the NS or N groups, green bars are taxa enriched in the P group, and blue bars are taxa enriched in the S group. Only taxa with an LDA value greater than 3.5 are shown. (I) *E. coli* loads in cecal chyme. Data were analyzed by one-way ANOVA and shown as means ± SEM (*n =* 6). Bars with different letters are significantly different among different groups. Group P = uninfected chicks; Group N = chicks treated with Nissle 1917 alone; Group S = *S*. Enteritidis infected chicks; Group NS = pretreated with Nissle 1917 + *S*. Enteritidis infected chicks. (J) Fold change of *S.* Enteritidis counts in the presence of Nissle 1917 in vitro. Data were analyzed by independent sample *t-*test and shown as means ± SEM (*n* = 6). Bars with different letters are significantly different between the 2 groups.

Alpha diversity analysis showed that the gavage of Nissle 1917 alone had no significant effect on the cecal microbial community richness and diversity of chicks. Pretreatment with Nissle 1917 limited the abnormally increased cecal microbial community richness and diversity caused by *S.* Enteritidis infection, as illustrated by ACE index, Chao1 index, Observed species, and Shannon index (Fig. 5A–D). Beta diversity analysis (PCoA and NMDS) and inter-group *P*-values of PERMANOVA, MRPP, ANOSIM, and AMOVA based on unweighted UniFrac indicated the cecum microbiota of chicks in group N was more similar to that in the P group. The cecum microbiota of chicks in the S and NS groups were separated from that of the P group (Fig. 5E and F, and Table 1). To further detail the differences of gut microbial composition among different groups, we performed taxonomic analyses of the intestinal microbiota among groups NS-vs-S-vs-P and groups N-vs-S-vs-P at different levels from phylum to genus by linear discriminant analysis effect size (LEfSe) (LDA >3.5). The results revealed that Enterobacteriaceae*, Escherichia-Shigella, E. coli,* and Lactobacillales were 4 common biomarkers of group N and group NS (Fig. 5G and H). *S.* Enteritidis infection increased the abundance of some opportunistic pathogens, such as *Clostridia_UCG_014, Flavonifractor,* and *Anaerotruncus*. Moreover, it is worth-noting that when compared with groups P and S, the abundance of beneficial bacteria, including Lactobacillales*,* Prevotellaceae*,* Lachnospiraceae*_*NK4A136_group*, Blautia, Bacteroides_fragilis, Lactobacillus_reuteri,* and *Lactobacillus_johnsonii*, was enriched in group NS. In addition, the number of *E. coli* recovered from the cecum chyme of chicks in the N and NS groups was significantly more than those of chicks in the S and P groups (*P* < 0.05; Fig. 5I). The co-culture assay verified that Nissle 1917 inhibited the growth of *S.* Enteritidis in vitro (*P* < 0.05; Fig. 5J).

**Table 1:** PERMANOVA, MRPP, ANOSIM, and AMOVA *P*-values based on the microbial community between groups (*n* = 6).

Item^1^	*P*-value				
	PERMANOVA	MRPP	ANOSIM	AMOVA (unweighted_unifrac)	AMOVA (weighted_unifrac)
P vs. N	0.350	0.360	0.541	0.141	0.254
P vs. S	0.004	0.008	0.005	0.003	0.012
P vs. NS	0.015	0.018	0.029	0.318	0.010
N vs. S	0.010	0.011	0.023	0.001	0.083
N vs. NS	0.105	0.091	0.140	0.021	0.005
S vs. NS	0.022	0.021	0.060	0.001	0.052

PERMANOVA = permutational multivariate analysis of variance; MRPP = multiple response permutation procedure; ANOSIM = analysis of similarities; AMOVA (unweighted_unifrac) = analysis of molecular variance based on unweighted_unifrac; AMOVA (weighted_unifrac) = analysis of molecular variance based on weighted_unifrac.

1 Group P = uninfected chicks; Group N = chicks treated with Nissle 1917 alone; Group S = *S*. Enteritidis infected chicks; Group NS = pretreated with Nissle 1917 + *S*. Enteritidis infected chicks.

## 4 Discussion

As a zoonotic bacterium, *S.* Enteritidis threatens global human and animal health. Antibiotics are the main treatments used to control *Salmonella* infection. However, it is well known that long-term and excessive use of antibiotics can lead to problems such as pathogen drug resistance and antibiotic residues (Ye et al., 2007). Probiotics are promising antibiotic substitutes, that can inhibit the growth and reproduction of pathogenic bacteria (Nakphaichit et al., 2019), regulate immunity (Huang et al., 2020), enhance intestinal barrier function, and promote the balance of intestinal microbiota (Zhang et al., 2021). Screening for probiotics that can inhibit *S.* Enteritidis is expected to eliminate the disadvantages of antibiotics. Nissle 1917, as the most well-known probiotic member of Enterobacteriaceae, has superior potential as an antibiotic substitute to against *S.* Enteritidis infection. Here, we systematically investigated the anti-*S.* Enteritidis effects of Nissle 1917 in chicks. Our results showed that challenging with *S.* Enteritidis decreased chick weight and increased the spleen index. Inoculation of Nissle 1917 before infection prevented weight loss and splenomegaly caused by *S.* Enteritidis infection. Pre-inoculation with Nissle 1917 significantly reduced *S.* Enteritidis loads in the liver, spleen, and cecum chyme by 9.33, 5.13, and 4.90, respectively. Liver histopathological changes analyzed by H&E staining also indicated that Nissle 1917 pretreatment significantly reduced pathological damage to the liver and reduced the inflammatory response caused by *S.* Enteritidis infection.

Inflammatory response plays an important role in host resistance to *Salmonella* infection. Certain levels of proinflammatory cytokines can stimulate macrophages to produce bacteriostatic substances (NO, etc.) to eliminate *Salmonella* (Nadeau et al., 2002; Shi et al., 2016); however, excessive accumulation of pro-inflammatory cytokines leads to inflammation (Gulig et al., 1997; Broz et al., 2012) and damage to the intestinal barrier (Beaurepaire et al., 2009). The inflammatory environment could promote replication and diffusion of *Salmonella* in the host (Spees et al., 2014; McLaughlin et al., 2019). Our results showed that intestinal proinflammatory factor (*TNF-α, IFN-γ* and *IL-8*) gene expression was up-regulated after *S.* Enteritidis infection. Nissle 1917 treatment prevented excessive up-regulation of proinflammatory factor gene expression induced by *S.* Enteritidis infection. These results are consistent with the results of previous studies in which Nissle 1917 reduced gene expression of proinflammatory factors *TNF-α* and *IFN-γ* in the cecum and reduced colonization of *Salmonella typhimurium* in mouse intestines (Deriu et al., 2013).

The intestinal barrier is the first line of defense between a host and the luminal environment. Pathogens in the intestine must cross the intestinal barrier before they can invade the internal organs of the host. Because Nissle 1917 significantly reduced *Salmonella* burden in the liver and spleen of infected chicks, we speculated that Nissle 1917 would improve intestinal health of the chicks. To verify this hypothesis, intestinal morphology and intestinal barrier function in the chick jejunum were determined. As expected, *Salmonella* infection decreased the ratio of VCR in the jejunum, which was partially alleviated by pretreatment of Nissle 1917. VCR plays an essential role in nutrient absorption and provides a protective barrier (Zhu et al., 2017). Lower VCR values may be one cause of weight loss in chicks challenged with *S.* Enteritidis. Previous study has found that *Salmonella* infection down-regulated tight junction gene expression and compromised the intestinal barrier in chicks (Zhang et al., 2020). Consistent with this, we confirmed that the *S.* Enteritidis challenge decreased the mRNA expression of claudin-1, occludin, *ZO-1,* and *MUC2* in the jejunum. ZO-1*,* occludin, and claudins are major intestinal barrier proteins (Yi et al., 2018). *MUC2* is involved in the secretion of intestinal mucin and increases the integrity of the intestinal barrier (Reynolds et al., 2020). Our data also demonstrated that oral administration of Nissle 1917 to *S.* Enteritidis infected chicks yielded mRNA expression of claudin-1, occludin, *ZO-1,* and *MUC2* in the jejunum which was similar to the uninfected chicks. These results reveal that probiotic Nissle 1917 can improve intestinal barrier function and reduce *S.* Enteritidis translocation from the intestine to visceral organs.

As previous reports stated that *ACE2*/*SL6A19* affect the microecological balance of the intestinal tract and regulate intestinal inflammation by regulating neutral amino acid transport (Kowalczuk et al., 2008; Camargo et al., 2009), and our previous study found that the *ACE2* gene was highly expressed in chicken intestinal tissues (Cong et al., 2021). We speculate that *ACE2* in the gut may mediate in the host inflammatory response induced by *S.* Enteritidis infection through the neutral amino acid homeostasis alterations. Our results showed that mRNA expression of *ACE2* and *SL6A19/B*^*0*^*AT1* in the cecum were significantly down-regulated by *S.* Enteritidis infection, but were partially rescued by pretreatment with Nissle 1917. Correspondingly, the content of neutral amino acids (Gly, Ser, Gln, and Trp) in serum was significantly reduced by *S.* Enteritidis infection while was restored by pretreatment with Nissle 1917. These results are consistent with research that found that *ACE2* gene expression of intestinal epithelial cells was significantly down-regulated under inflammatory conditions (Burgueño et al., 2020). Another study also reported that activating the ACE2 pathway could inhibit intestinal inflammation induced by stress (Yisireyili et al., 2018).

Previous study has confirmed that *S.* Enteritidis infection will lead to an imbalance of the intestinal flora in chicks (Juricova et al., 2013). In addition, the works of Hashimoto et al. (2012) further confirmed that the increased susceptibility of *ACE2* knockout mice to intestinal inflammatory diseases is related to an imbalance of the intestinal flora composition. As the chicken gut microbiome is most susceptible to interventions early in life (Nurmi and Rantala, 1973; Jurburg et al., 2019), we pretreated chicks with Nissle 1917 at the 4 d to 6 d after hatch and investigated the effects of Nissle 1917 on cecum microbiota composition of chicks infected vs. uninfected with *S.* Enteritidis. Our results indicated that gavage of Nissle 1917 alone has no significant effect on the cecal microbial community richness and diversity of chicks; however, challenging with *S.* Enteritidis abnormally increases cecal microbial community richness and diversity, which is partially reversed by pretreatment with Nissle 1917. The cecum microbiota of chicks in the N group was more similar to that in the P group, whereas the cecum microbiota of chicks in the S and NS groups were separated from that of the P group. Taxonomic analyses by LEfSe showed that *S.* Enteritidis infection increased the abundance of some opportunistic pathogens, such as *Clostridia_UCG_014, Flavonifractor,* and *Anaerotruncus*. *Clostridia_UCG_014* is a proinflammatory bacteria involved in the inflammatory responses (Wang et al., 2021). Inflammation can result in the production of tetrathionate and other metabolites, thereby enhancing *Salmonella* growth and improving its persistence and spread (Winter et al., 2010; Thiennimitr et al., 2011). *Flavonifractor* is a member of the resident gut microbiota but has been shown to cause infection in an immunocompromised patient (Berger et al., 2018), and oral administration of *Flavonifractor* suppresses the Th2 immune response in mice (Ogita et al., 2020). *Anaerotruncus colihominis* has been reported to cause bacteremia (Lau et al., 2006). By contrast, in the current study, the gavage of Nissle 1917 increased the abundance of beneficial Lactobacillales*,* Prevotellaceae*,* Lachnospiraceae*_*NK4A136_group*, Blautia, Bacteroides_fragilis, Lactobacillus_reuteri,* and *Lactobacillus_johnsonii*. Most of these enriched bacteria have been proved to limit the colonization and translocation of *Salmonella*, or attenuate *Salmonella* pathogenicity, thus protect the host from *Salmonella* infection, such as *Lactobacillus* (Arreguin-Nava et al., 2019), Lachnospiraceae (Antunes et al., 2014; García et al., 2017), *Bacteroides_fragilis* (Vernay et al., 2020), *Blautia* (Verstraeten et al., 2022), *Lactobacillus_johnsonii* (Yang et al., 2022; Olnood et al., 2015), and *Lactobacillus_reuteri* (Nakphaichit et al., 2019; Shi et al., 2019, 2022). In addition, *Lactobacillus* has been used as probiotics with beneficial effects on poultry performance (Angelakis and Raoult, 2010) and immunity via modulation of the intestinal microbiome (Forte et al., 2016; Thakur et al., 2016). Prevotellaceae is related to carbohydrate and lipid metabolism, showed direct correlations with body weight (Qiu et al., 2022). Increased abundance of Lachnospiraceae was reported to relate to colonization resistance to *Campylobacter* infection (Kampmann et al., 2016). As a family of anaerobic, spore-forming bacteria, Lachnospiraceae are known to degrade complex polysaccharides to short-chain fatty acids (Drennan et al., 2016). Short-chain fatty acids directly inhibit pathogen growth in vitro by disrupting intracellular pH homeostasis and protect mice from *S. typhimurium* (Jacobson et al., 2018).

## 5 Conclusion

In conclusion, the gavage of Nissle 1917 helps chicks resist *S.* Enteritidis infection and reshape the cecal microbiota composition. In addition, we found that the anti-*S.* Enteritidis effect of Nissle 1917 is mediated by the ACE2-SLC6A19 pathway. Our work highlights that *E. coli* Nissle 1917 shows great potential to replace antibiotics in clinical therapy as well as in the animal and food industry to fight *S.* Enteritidis infection. Additionally, the ACE2-SLC6A19 pathway maybe an effective target for regulating pathogen infection.

## Author Contributions

**Shu Wu** and **Qianyun Zhang:** Conceptualization, Data curation, Formal analysis, Investigation, Methodology, Project administration, Software, Visualization and Writing - Original draft. **Guanglei Cong:** Conceptualization, Data curation, Investigation, Methodology, Software and Visualization. **Yunqi Xiao:** Conceptualization, Data curation, Formal analysis, Methodology and Validation. **Yiru Shen** and **Shan Zhang:** Data curation, Funding acquisition and Investigation. **Wenchang Zhao:** Investigation. **Shourong Shi:** Funding acquisition, Resources, Supervision and Writing - Review & Editing. All authors have read and agreed to the published version of the manuscript.

## Data availability

These sequence data have been submitted to the Biotechnology Information (NCBI) Sequence Read Archieve databases under accession number PRJNA780678.

## Declaration of competing interest

We declare that we have no financial and personal relationships with other people or organizations that can inappropriately influence our work, and there is no professional or other personal interest of any nature or kind in any product, service and/or company that could be construed as influencing the content of this paper.
