# Supplementary material for: Probiotic Escherichia coli Nissle 1917 protect chicks from damage caused by Salmonella enterica serovar Enteritidis colonization
Source: Anim Nutr. 2023 Jun 8;14:450–60. doi: 10.1016/j.aninu.2023.06.001 (PMC10463197; doi:10.1016/j.aninu.2023.06.001)
Supplement: Multimedia component 1 [file mmc1.docx]

**Supplementary material**

**Graphic abstract**

**
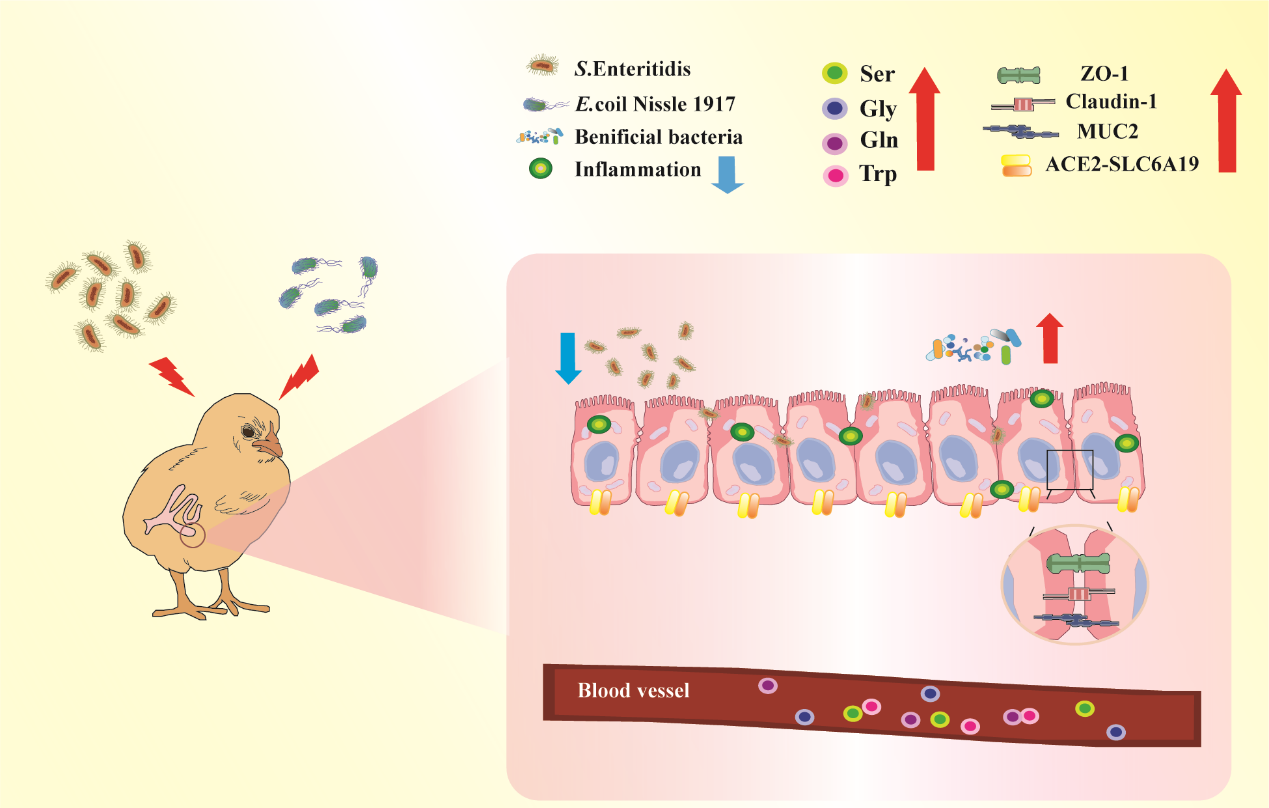
**


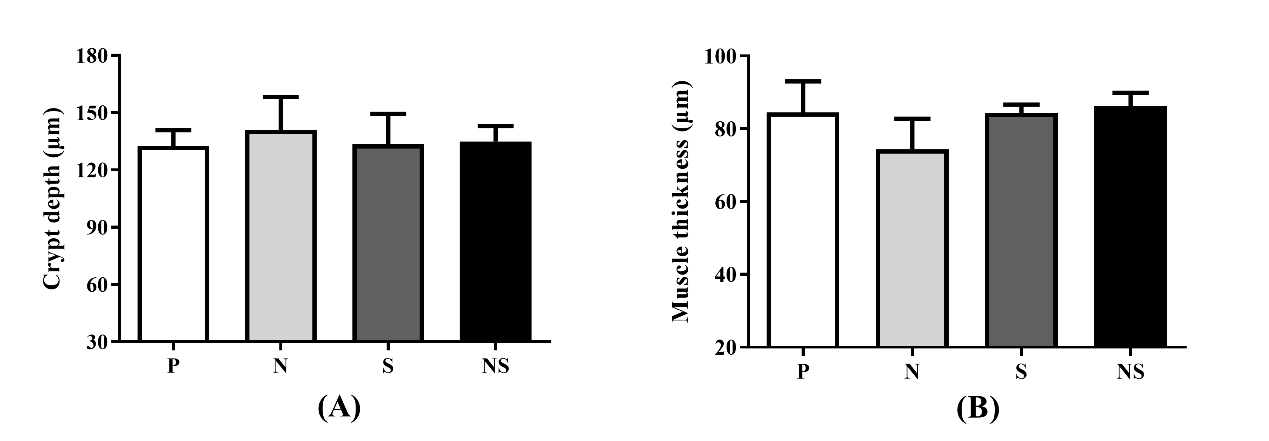


**Fig. S1** Effects of Nissle 1917 on the jejunum morphology of *S.* Enteritidis infected or uninfected chicks. (A) Crypt depth. (B) Muscle thickness. Group P = uninfected chicks; Group N = chicks treated with Nissle 1917 alone; Group S = S. Enteritidis infected chicks; Group NS = pretreated with Nissle 1917 + S. Enteritidis infected chicks. Data were tested by one-way ANOVA and shown as means ± SEM (*n* = 6).

**Table S1** Diet composition and nutrient levels from 1 to 9 d of age (as-fed basis, %).

| **Ingredients** | **Content** |
| --- | --- |
| Corn | 55.24 |
| Soybean meal, 46% | 36.92 |
| Soybean oil | 3.50 |
| Limestone | 1.12 |
| Calcium hydrogen phosphate | 2.10 |
| Methionine | 0.28 |
| Lysine, 98% | 0.22 |
| NaCl | 0.30 |
| Vitamin premix^1^ | 0.03 |
| Mineral premix^2^ | 0.20 |
| Choline chloride, 70% | 0.09 |
| Total | 100.00 |
| **Nutrient levels**^3^**, %** |  |
| ME, kcal/kg | 2950 |
| Crude protein | 21.00 |
| Calcium | 1.00 |
| Total phosphorus | 0.67 |
| Nonphytate phosphorous | 0.45 |
| Digestible Lys | 1.20 |
| Digestible sulfur-containing amino acid | 0.85 |
| Digestible Thr | 0.66 |
| Digestible Trp | 0.22 |

^1^ Premix vitamin provided per kilogram of diet: Vitamin A (retinyl palmitate), 8000 IU; vitamin D3 (cholecalciferol), 1000 IU; vitamin E (D, L-α-tocopheryl acetate), 20 IU; vitamin K3 (menadione sodium bisulfate complex), 0.50 mg; vitamin B1, 2.00 mg; vitamin B2, 8.00 mg; vitamin B6, 3.50 mg; vitamin B12 (cobalamin), 10.00 μg; niacin, 35.00 mg; calcium pantothenic, 10.00 mg; folic acid, 0.55 mg; biotin, 0.18 mg.

^2^ Premix mineral provided per kilogram of diet: Fe, 80.00 mg; Mn, 100.00 mg; Zn, 80.00 mg; I, 0.70 mg; Se, 0.30 mg; Cu, 8.00 mg.

^3^ ME was a calculated value was calculated according to feed composition according to NRC (1994), whereas the other nutrient levels were measured values according to AOAC (2016).

**Table S2** Gene-specific primers for related genes.

| **Gene** | **GenBank accession no.** | **Primer Orientation** | **Primer sequence (5′→3′)** | **Product size, bp** |
| --- | --- | --- | --- | --- |
| *GAPDH* | NM_204305.1 | Forward | GCCCAGAACATCATCCCA | 137 |
|  |  | Reverse | CGGCAGGTCAGGTCAACA |  |
| *NOS2* | NM_204961.1 | Forward | CCTGGAGGTCCTGGAAGAGT | 82 |
|  |  | Reverse | CCTGGGTTTCAGAAGTGGC |  |
| *TNFA* | NM_204267.1 | Forward | CAGGACAGCCTATGCCAACAAG | 114 |
|  |  | Reverse | GGTTACAGGAAGGGCAACTCATC |  |
| *IFNG* | NM_205149.1 | Forward | CAAGCTCCCGATGAACGACTT | 162 |
|  |  | Reverse | AGTTGAGCACAGGAGGTCAT |  |
| *IL1B* | NM_204524.1 | Forward | CCGAGGAGCAGGGACTTT | 133 |
|  |  | Reverse | AGGACTGTGAGCGGGTGT |  |
| *IL6* | NM_204628.1 | Forward | TTTATGGAGAAGACCGTGAGG | 106 |
|  |  | Reverse | TGTGGCAGATTGGTAACAGAG |  |
| *IL8* | NM_205498.1 | Forward | ATGAACGGCAAGCTTGGAGCTG | 233 |
|  |  | Reverse | TCCAAGCACACCTCTCTTCCATCC |  |
| Claudin-1 | NM_001013611 | Forward | CTGATTGCTTCCAACCAG | 140 |
|  |  | Reverse | CAGGTCAAACAGAGGTACAAG |  |
| Occludin | NM_205128.1 | Forward | TCATCGCCTCCATCGTCTAC | 141 |
|  |  | Reverse | TCTTACTGCGCGTCTTCTGG |  |
| *ZO-1* | XM_413773 | Forward | CTTCAGGTGTTTCTCTTCCTCCTC | 131 |
|  |  | Reverse | CTGTGGTTTCATGGCTGGATC |  |
| *MUC2* | NM_001318434.1 | Forward | GTGAAGACCCTGATGAAA | 219 |
|  |  | Reverse | GTGAACACTGGCGAGAAT |  |
| *ACE2* | XM-416822.5 | Forward | TGGAGGTGGATGGTGTTT | 107 |
|  |  | Reverse | TCGTGAGGGACTGGTTCG |  |
| *SLC6A19* | XM_419056.6 | Forward | TATCCTGGCTGGGTCTATGC | 125 |
|  |  | Reverse | AGGCCTGTACGATCCCTTCT |  |

*GAPDH* = glyceraldehyde-3-phosphate dehydrogenase; *NOS2* = nitric oxide synthase; *TNF-α* = tumor necrosis factor-alpha; *IFN-γ* = interferon-gamma; *IL-1β* =interleukin 1 beta; *ZO-1*= zonula; *MUC2* = mucin 2; *ACE2* = angiotensin-converting enzyme II; *SLC6A19* = solute carrier family 6 member 19.

**References**

AOAC. Official Methods of Analysis of AOAC International. 20th ed. 2016.

NRC. Nutrient requirements of poultry. 9th ed. Washington, DC, USA: National Academy Press; 1994.
